# National Surveillance of Enterovirus D68 Upsurge, France, 2024

**DOI:** 10.3201/eid3207.260044

**Published:** 2026-07

**Authors:** Marion Jeannoël, Maxime Bisseux, Stéphanie Dan, Elisa Creuzet, Delphine Parraud, Jean-Luc Bailly, Jérémie Lebeurre, Amélie Brebion, Hélène Chabrolles, Laurence Josset, Cécile Henquell, Isabelle Schuffenecker, Audrey Mirand, Enterovirus Surveillance Network

**Affiliations:** Hospices Civils de Lyon, Centre National de Référence des Entérovirus–Laboratoire Associé, Lyon, France (M. Jeannoël, S. Dan, D. Parraud, J. Lebeurre, L. Josset, I. Schuffenecker); Université Clermont Auvergne, Clermont-Ferrand (M. Bisseux, E. Creuzet, H. Chabrolles, C. Henquell, A. Mirand); Centre Hospitalier Universitaire de Clermont-Ferrand, Centre National de Référence des Entérovirus–Laboratoire Coordonnateur, Clermont-Ferrand, France (M. Bisseux, E. Creuzet, A. Brebion, H. Chabrolles, C. Henquell, A. Mirand); Université Claude Bernard Lyon 1, Villeurbanne, France (S. Dan, D. Parraud, L. Josset); Université Clermont Auvergne, Clermont-Ferrand (J.-Lo Bailly)

**Keywords:** enterovirus D68, viruses, respiratory infections, molecular epidemiology, epidemiologic surveillance, disease outbreak, enterovirus infections, myelitis, France

## Abstract

In 2024, an early and rapid rise in enterovirus D68 (EV-D68) infections in France prompted the implementation of enhanced nationwide surveillance to characterize the outbreak. EV-D68 screening was performed as part of the routine hospital strategy of the 2 national reference laboratories and of the national surveillance of enterovirus infections. Of 919 patients, 49.1% (451/919) were adults. Severe infection was reported in 169 patients (102 children and 67 adults). We observed neurologic complications in 7 children (seizures and encephalitis) and 4 adults (myelitis). Infections peaked in week 38 and were associated with subgenotypes A2 and B3; A2 predominated, particularly in adults (317/457 [69.3%] A2 infections). Complete genome analyses identified a new A2-derived lineage with mutations clustering in exposed regions of viral capsid protein 1. Our findings highlight the substantial clinical impact of EV-D68 in adults as well as children, underscoring the need for broad clinical and genomic surveillance.

Although enterovirus D68 (EV-D68), first isolated in 1962, has been considered an emerging pathogen since 2008 (*1*), it attracted attention after the US Centers for Disease Control and Prevention (CDC) issued alerts in 2014 (*2*), which were followed by reports of numerous cases worldwide (*3*). EV-D68 causes severe respiratory infections in children and adults, particularly in patients with underlying respiratory disease (*3*,*4*). Neurologic complications can occur, and some constitute a specific neurologic entity, acute flaccid myelitis (AFM), characterized by frequent sequelae (*5*). Several countries have thus strengthened existing surveillance of enterovirus infections by combining it with respiratory, syndromic (asthma-like illness, AFM, or both) or even environmental surveillance (*6*–*9*). After markedly reduced circulation in 2020, EV-D68 infections resurged in autumn 2021, 2022, and 2023 in Europe (*4*,*8*,*10*–*12*) and in 2022 across the United States with increase in respiratory illness in pediatric patients (*13*). More recently, enhanced genomic surveillance evidenced EV-D68 upsurges in 2024 in Italy (*9*), Spain (*14*), and the United States (*15*). 

To date, EV-D68 strains are divided into 3 genotypes and 5 subgenotypes, designated A (A1 and A2, also referred to as clade D in some studies), B (B1–B3), and C. Since 2017, EV-D68 epidemics have been associated with the cocirculation of the subgenotypes B3 and A2 on all continents (*16*). In France, enterovirus surveillance involves a network of 32–36 hospital virology laboratories, the Enterovirus Surveillance Network (ESN), including the 2 enterovirus national reference laboratories (NRLs), based in Clermont-Ferrand (University Hospital of Clermont-Ferrand, France, coordinator) and in Lyon (Hospices Civils de Lyon, France, associate). Since the reemergence of EV-D68 in 2014, network members are regularly encouraged to screen respiratory specimens for EV-D68 at least in patients with severe infection (*17*). Such screening enabled the detection of EV-D68 epidemics in 2014, 2016, and 2018 (*4*,*17*,*18*), and then in 2021, 2022, and 2024 (*19*). Here, we describe the 2024 EV-D68 upsurge in France characterized by early circulation, detected as early as June, as well its effect on the adult population, and the cocirculation of B3 and A2 subgenotypes, including a new A2-derived lineage.

## Methods

### Patient Population, Study Design and Molecular Testing of Clinical Specimen*s*

Respiratory specimens were tested for EV-D68 either by an EV-D68 real-time reverse transcription PCR or by sequencing the viral protein (VP) 4/VP2 coding region (*20*–*22*). EV-D68 testing was done as part of the routine hospital diagnostic and genotyping strategies for enterovirus/rhinovirus-positive respiratory specimens from patients of all ages and year-round at NRL1, or for all respiratory specimens from children <5 years of age during September–November at NRL2. Both NRLs also analyzed enterovirus-positive samples from the ESN throughout the year, regardless of the sample type. The observation of early circulation and the increased detection of EV-D68 cases prompted the NRLs to implement an enhanced EV-D68 surveillance nationwide in France during September 2–December 1, 2024 (weeks 36–48). Enhanced surveillance consisted of extended EV-D68 screening to children <10 years of age starting week 36 in NRL2 and asking the ESN to retroactively and prospectively send enterovirus- or rhinovirus-positive respiratory specimens for EV-D68 screening by prioritizing samples from patients with severe infection. To determine whether the 2024 upsurge displayed distinct epidemiologic features compared with previous years, we compared the EV-D68 positivity rate of samples in 2024 with those in 2021–2023 (weeks 36–48) in both NRLs, whose strategies remained unchanged during 2021–2024. We considered 3 age groups: children <5 years of age (separated data for each NRL), children 5–17 years of age (data for NRL1), and adults >18 years of age (data for NLR1).

### Patients and Clinical Characteristics

We retrospectively collected demographic and clinical characteristics from medical charts. Severity criteria included admission to intensive care when not attributable to another cause, respiratory disease requiring intravenous corticosteroids, high-flow oxygen therapy or intubation, encephalitis, complex seizures, myelitis, and death, including sudden infant death. The ethics committee of Centre Hospitalier Universitaire de Clermont Ferrand approved this study (IRB00013412; CHU de Clermont Ferrand IRB #1, IRB no. 2024-CF393).

### VP1 and Complete Enterovirus Genome Sequencing

We determined the complete or partial VP1 sequences of EV-D68–positive specimens by in-house gene amplification and Sanger sequencing to assign a phylogenetic subgenotype (*4*). We compared the proportions of the different subgenotypes identified nationally among children and adults in 2024 with those of the 2016–2023 period. We performed whole-genome sequencing by next-generation sequencing (NGS) methods on a subset of samples representing the full genetic diversity observed in the 1D sequences (encoding the VP1 capsid protein) in 2021–2024, either after amplification of full-length genome (at NRL1) or using a metatranscriptomic approach (at NRL2) (*23*) (Appendix). We deposited all complete genome sequences in GenBank (accession nos. PQ612496–575, PP947790–800, and PP548243–8).

### Data Collection, Sequence Processing, and Bayesian Inference

As of June 1, 2025, we retrieved all available EV-D68 sequences >6,000-nt long that were deposited in GenBank, yielding a total of 1,813 sequences, including the 97 sequences obtained in this study from 2021–2024. After filtering steps (i.e., exclusion of sequences <6,495 nt and clusters of sequences collected in the same country and year that displayed <0.5% genetic differences), we obtained a final alignment comprising 449 high-quality EV-D68 sequences, including 42 study sequences (2024, n = 19; 2023, n = 8; 2022, n = 13, 2021, n = 2). We analyzed Bayesian inference using an uncorrelated lognormal relaxed molecular clock model to accommodate rate variation among lineages. We used the coalescent Bayesian Skyline model as the tree before accounting for demographic history. We modeled nucleotide substitutions using the general time reversible substitution model. We jointly estimated phylogenetic parameters through a Markov chain Monte Carlo process that ran for 500 million generations.

### Complete Genome and P1 Region Amino Acid Sequence Analyses

We compared the consensus of complete coding sequences indicating the predominant amino acids between historical strains A2-I and B3-I and the 2024 study strains A2-II and B3-II. We determined the positions of the mutations by aligning sequences with the prototype sequence (GenBank accession no. AY426531; Fermon strain). We predicted the 3-dimensional pentameric structure of A2 strains from 5 copies of the VP1–VP4 protein sequences using AlphaFold (https://alphafoldserver.com).

### Statistical Analysis

We conducted group comparisons by Fisher exact test; p<0.05 indicated a statistically significant difference. We used GraphPad Prism version 8.0.2 (GraphPad Software, https://www.graphpad.com) for all analyses.

## Results

### Epidemiologic Characteristics of the 2024 EV-D68 Epidemic

**Figure 1:**
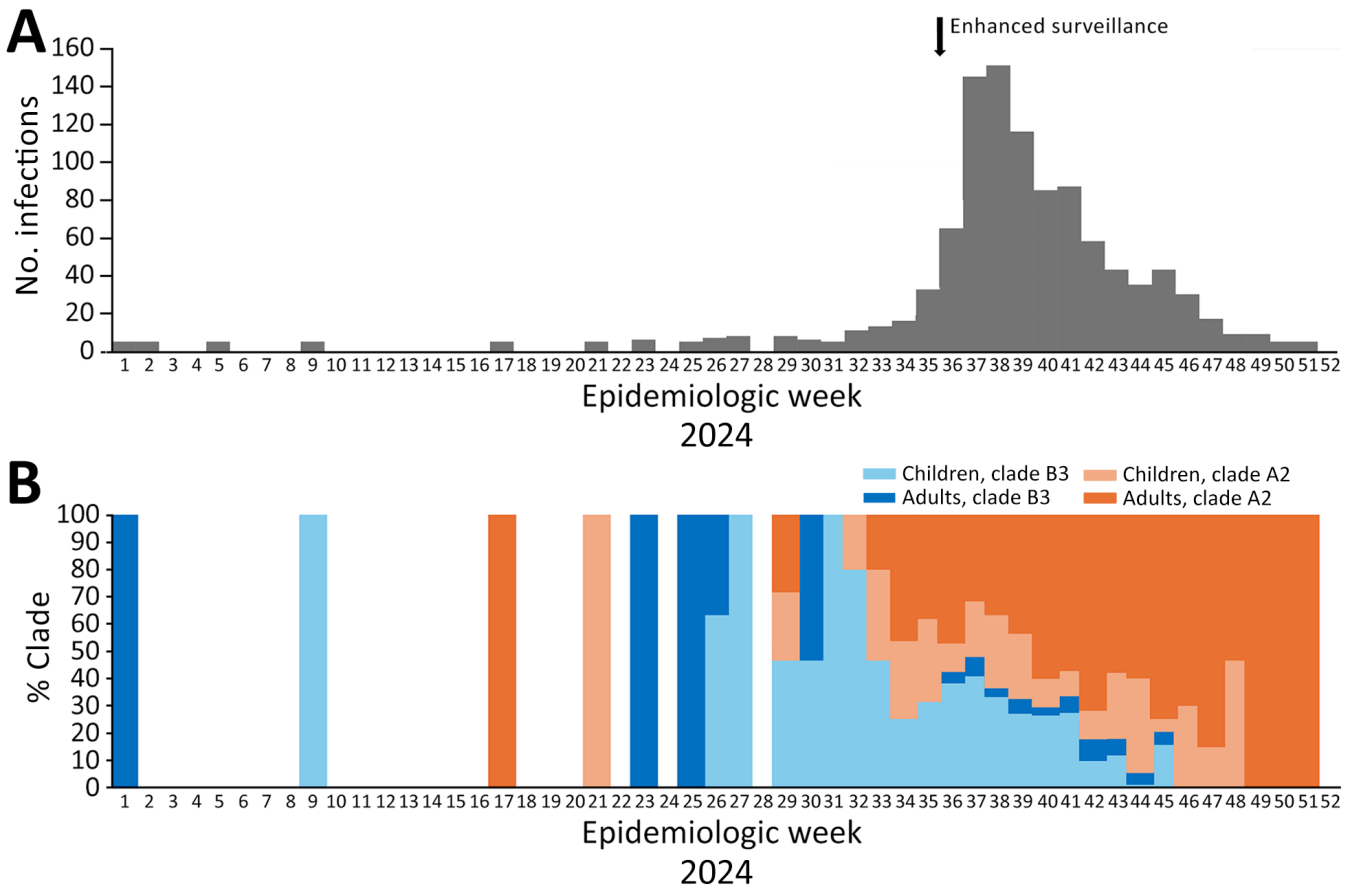
Distribution of infections from report on national surveillance of enterovirus D68 upsurge, France, 2024. A) Weekly distribution of infections in 2024. B) Distribution of subgenotype percentages. Each genotype is represented by a color, the intensity of which indicates the age group (children <18 years of age, adults >18 years of age).

**Figure 2:**
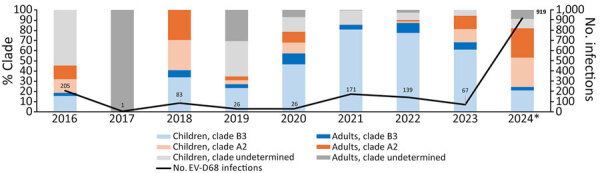
Distribution of EV-D68 subgenotype infections by year from report on national surveillance of enterovirus D68 upsurge, France, 2016–2024. Curve shows total number of infections per year. Children are defined as patients <18 years of age, adults >18 years of age. EV-D68, enterovirus D68.

We detected a total of 919 EV-D68 infections, 468 (50.9%) of which occurred in children. The EV-D68 epidemic peaked in week 38; the first consecutive weekly seasonal cases were detected starting week 29 (Figure 1, panel A). The age distribution of EV-D68 infections shifted over time. Whereas 332/532 (60.7%) samples collected during weeks 26–39 were from children, 247/378 (65.3%) samples collected from week 39 onward were from adults. Clade subgenotyping was possible for 721 (78.5%) of the 919 samples, among which 367 were from children and 354 from adults. Subgenotypes B3 and A2 cocirculated; A2 was predominant in 457/721 (63.3%) samples. Subgenotype B3 was more frequently detected in children (227/367 [61.8%]) than in adults (37/354 [10.5%]; p<0.0001); adults were more frequently infected with subgenotype A2 (317/354 [89.5%]) (Figure 1, panel B; Appendix Table). Subgenotype A2 became predominant from week 34, accounting for 64% of samples during weeks 34–48, and seemed to expand again after 5 years of B3 predominance (Figure 2). 

**Figure 3:**
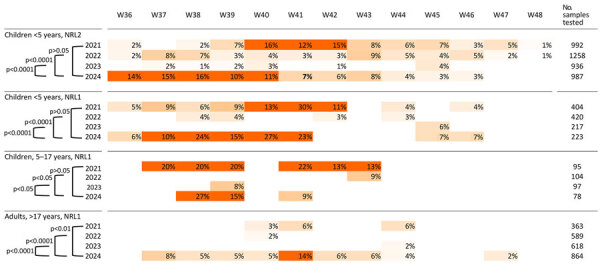
Percentages of positive samples, by week and by age group, from report on national surveillance of enterovirus D68 upsurge, France, 2021–2024. Data are based on routine hospital strategies of each of the 2 NRLs. To determine whether the 2024 upsurge had distinct epidemiologic features compared with previous years, we compared the positivity rate of samples in 2024 with those in 2021–2023 (weeks 36–48) in both NRLs. We included 3 age groups: children <5 years of age (data for both NRLs), children 5–17 years of age (data only for NRL1), and adults >17 years old (data only for NRL1). NRL, national reference laboratory; W, week.

To further characterize the upsurge, we compared the EV-D68 positivity rate of samples collected during weeks 36–48 of 2021–2024, exclusively using data from the routine hospital activity of the NRLs whose strategies have remained unchanged since 2021 (Figure 3). Among children <5 years of age, we observed a significant increase in EV-D68 prevalence in 2024 compared with 2022 and 2023, regardless of the testing strategy in each NRL. In 2024, a sustained circulation (>10% positivity rate) was documented earlier and over a longer period than in 2021, at 6 weeks versus 3 weeks. In the adult population, the 2024 epidemic was characterized by a higher positivity rate (2021–2024, p<0.01; 2022–2024 and 2023–2024, p<0.0001) and a broader temporal amplitude than in previous years.

### Demographic and Clinical Characteristics of EV-D68 Infections

Clinical data were available for 668/919 patients with EV-D68 infections. We excluded 16 patients, 7 children and 9 adults, because EV-D68 was considered unlikely on the basis of their clinical symptoms. We analyzed demographics, clinical outcomes (Table 1), and clinical characteristics (Table 2) for the 652 remaining patients. A total of 308/358 children and 253/281 adults required hospitalization; of the adults, 126/150 were 18–64 years of age and 127/131 >65 years of age. Hospitalization rate was significantly higher in adults >65 years of age (96.9%) than in children (86.0%; p = 0.0003) and than in adults 18–64 years of age (84.0%; p = 0.0002) (Table 1). Intensive care was needed for 85/343 children and 69/268 adults; of the adults, 42/143 were 18–64 years of age and 27/125 were >65 years of age. Infections met severity criteria in 102/363 (28.1%) children and 67/289 (23.2%) adults. We observed no association between clade and severity. 

**Table 1:** Patient characteristics and clinical outcomes in study of EV-D68 upsurge, France, 2024*

Characteristic	Children 0–17 y, n = 363	Adults		
		18–64 y, n = 155	>65 y, n = 134	Total, n = 289
Median age, y (IQR)	2.0 (0.04–7)	45 (34–56)	76 (70–82)	62 (43–75)
Female/male ratio	0.76	0.49	0.55	1.08
EV-D68 severe infections	102/363 (28.1)	38/155 (24.5)	29/134 (21.6)	67/289 (23.2)
Hospitalization†	308/358 (86.0)	126/150 (84.0)	127/131 (96.9)	253/281 (90.04)
Intensive care unit‡	85/343 (24.78)	42/143 (29.4)	27/125 (21.6)	69/268 (25.75)
Death	2/363 (0.6)§	3/155 (1.9)	6/134 (4.5)	9/289 (3.1)¶

*Values are no. (%) except as indicated. EV-D68, enterovirus D68; IQR, interquartile range. †Hospitalization requirement was reported for 358 children and 281 adults. ‡Intensive care requirement was reported for 343 children and 268 adults. §Sudden infant death syndrome. Although EV-D68 was the only detected pathogen, EV-D68 infection was considered as the possible cause of death; attributing causality can be challenging without thorough investigation in such contexts. ¶Among the 9 adults, the EV-D68 infection was considered as the probable cause of death in 4 patients (EV-D68 was the only detected pathogen) and as possible in 5 patients (evidence or strong suspicion of bacterial co-infection).

**Table 2:** Clinical characteristics of cases in study of enterovirus D68 upsurge, France, 2024*

Characteristic	Children 0–17 y, n = 363			Adults >18 y, n = 289			Total, N = 652	
	No. (%) patients	No. (%) severity criteria		No. (%) patients	No. (%) severity criteria		No. (%) patients	No. (%) severity criteria
Respiratory symptoms	332 (91.5)	91 (25.1)		278 (96.2)	55 (19.0)		610 (93.6)	146 (22.4)
URTI	100 (27.5)	3 (0.8)		81 (28.0)	0		181 (27.8)	3 (0.5)
Asthma	147 (40.5)	61 (16.8)		33 (11.4)	11 (3.8)		180 (27.6)	72 (11.0)
Bronchiolitis, bronchitis, bronchial syndrome	45 (12.4)	12 (3.3)		24 (8.3)	0		69 (10.6)	12 (1.8)
Pneumonia	18 (5.0)	4 (1.1)		52 (18.0)	15 (5.2)		70 (10.7)	19 (2.9)
Dyspnea	5 (1.4)	0		15 (5.2)	0		20 (3.1)	0
Neonatal apnea/distress	3 (0.8)	0		NA	NA		3 (0.5)	0
Acute exacerbation of COPD	NA	NA		41 (14.2)	12 (4.2)		41 (6.3)	12 (1.8)
Respiratory distress	15 (4.1)	7 (1.9)		31 (10.7)	17 (5.9)		46 (7.1)	24 (3.7)
Neurologic symptoms	17 (4.7)	7 (1.9)		5 (1.7)	4 (1.4)		22 (3.4)	11 (1.7)
Seizures	14 (3.9)	7 (1.9)		1 (0.3)	0		15 (2.3)	7 (1.1)
Encephalitis	4 (1.1)	4 (1.1)		0	0		4 (0.6)	4 (0.6)
Meningitis	1 (0.3)	0		0	0		1 (0.2)	0
Myelitis, Guillain-Barré syndrome	0	0		4 (1.4)	4 (1.4)		4 (0.6)	4 (0.6)
Other	1 (0.3)†	0		0	0		1 (0.2)	0
Cardiovascular symptoms	2 (0.6)	2 (0.6)		21 (7.3)	14 (4.8)		23 (3.5)	16 (2.5)
Decompensated heart failure	1 (0.3)	1 (0.3)		15 (5.2)	7 (2.4)		16 (2.5)	8 (1.2)
Myocarditis	1 (0.3)	1 (0.3)		0	0		1 (0.2)	1 (0.2)
Sepsis, shock	0	0		12 (4.2)	11 (3.8)		12 (1.8)	11 (1.7)
Enteric symptoms	57 (15.7)‡	0		25 (8.7)‡	0		82 (12.6)	0
Isolated fever	6 (1.7)	0		7 (2.4)	0		13 (2.0)	0

*A given patient may experience >1 clinical item. COPD, chronic obstruction pulmonary disease; NA, not applicable; URTI, upper respiratory tract infection. †Irritability and hypotonia. ‡Acute gastroenteric symptoms were the only clinical signs in 9 children and 2 adults.

Respiratory symptoms were predominant in both children and adults. Eleven patients (1.7%) experienced severe neurologic symptoms associated with respiratory signs. Although none of the children experienced AFM, 4 adults were hospitalized with myelitis, 1 case of facial paralysis with left motor deficit and 3 cases of Guillain-Barré syndrome (GBS). All 3 GBS patients experienced abolished osteotendinous reflexes and rapidly progressive motor paralysis, followed by acute respiratory failure requiring intensive care. GBS was documented on electroneuromyography for 2 patients. EV-D68 was the only pathogen detected in 2 patients with GBS and was associated with multiple pathogens in the third patient. All adult neurologic cases were associated with subgenotype A2 infection.

### Phylogenetic and Amino Acid Variation Analyses

**Figure 4:**
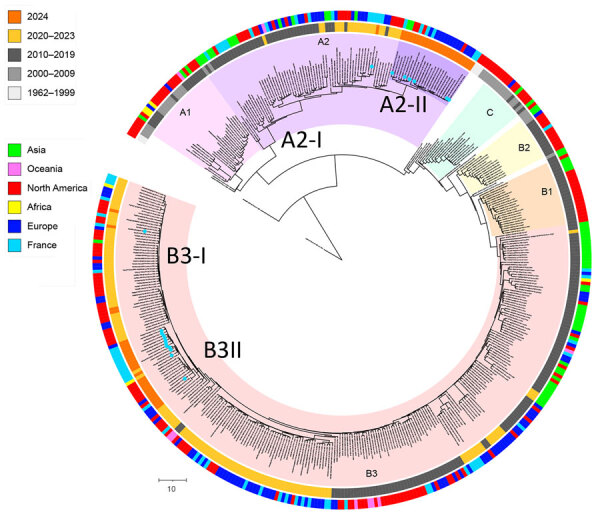
Time-scaled phylogeny of enterovirus D68 from report on national surveillance of enterovirus D68 upsurge, France, 2024. We reconstructed a time-calibrated phylogenetic tree based on all available complete or near-complete (<6,495 nt) ORF sequences retrieved from GenBank, along with the sequences generated in this study. We subsampled and analyzed a total of 449 sequences using Bayesian inference with BEAST version 2.6.3 (https://beast.community), running a Markov chain of 500 million generations. We summarized the maximum clade credibility tree using TreeAnnotator (https://beast.community/treeannotator), and visualized with iTOL (https://itol.embl.de). We added 2 outer annotation rings, the outer ring for the continent or country of origin and the inner ring for the year of sampling. Previously defined clades (A1, A2, B1–B3, and C) are labeled and highlighted for branches and taxa: pink for clade A1, light purple for A2 and darker purple for sequences from the newly identified 2024 A2 subgroup A2-II, light orange for B1, yellow for B2, peach for B3, and light green for C. We identified the 2 groups of B3 sequences in this study as B3-I (ancestral) and B3-II. Blue dots indicated at the taxon names indicate sequences generated in this study in 2024. Scale bar represents the divergence in years relative to 2024.

**Figure 5:**
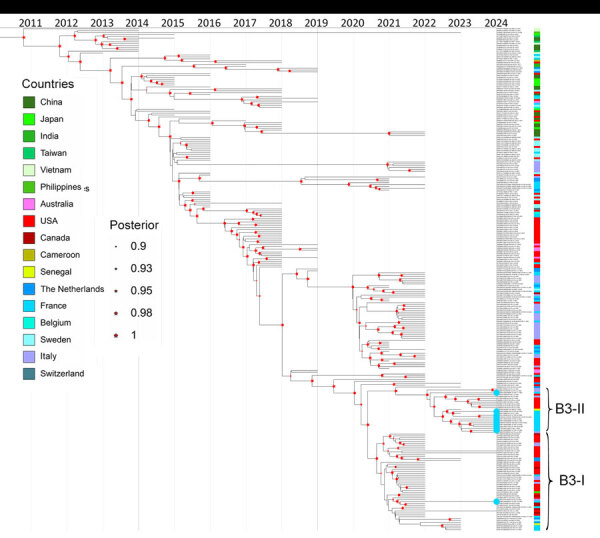
Bayesian time-scaled phylogeny of enterovirus D68, subgenotype B3, from report on national surveillance of enterovirus D68 upsurge, France, 2021–2024. Phylogeny created using BEAST version 2.6.3 (https://beast.community). A subtree focusing on the study strains belonging to subgenotype B3 was extracted from the complete time-calibrated phylogenetic tree of EV-D68. The estimated ages of the most recent common ancestors from the root of each clade to the year 2024 are indicated. Red stars at nodes indicate posterior probabilities >0.9, star sizes are proportional to posterior probability (larger symbols indicate values closer to 1). Colors at right indicate country of origin for each strain. Blue dots indicated at the taxon names indicate sequences generated in this national study in 2024. GenBank accession numbers are provided.

**Figure 6:**
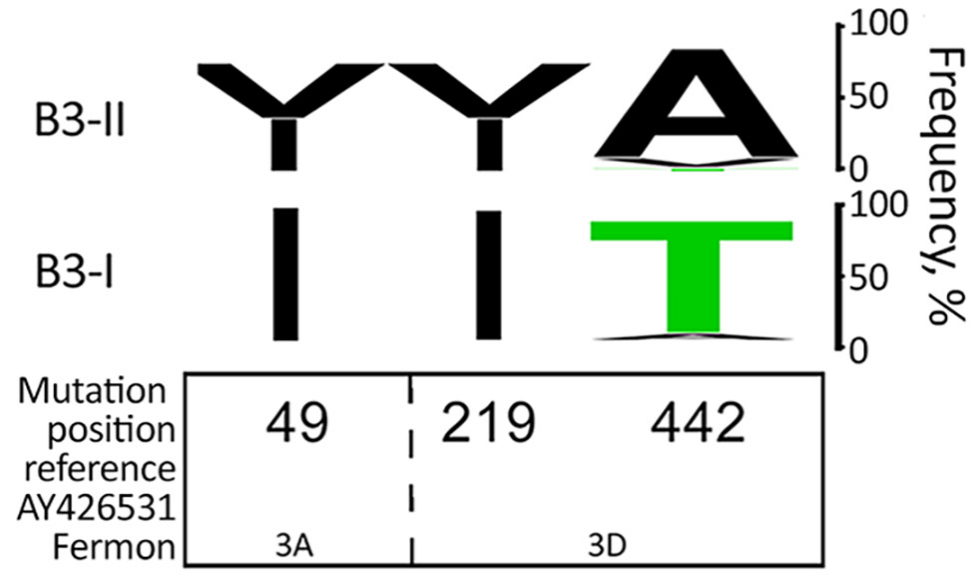
Amino acid change analysis of enterovirus D68, subgenotype B3, from report on national surveillance of enterovirus D68 upsurge, France, 2024. Amino acid substitutions leading to changes in the predominant residues between B3-II (n = 61 sequences) and the monophyletic clade corresponding to the nearest common ancestor B3-I (n = 106 sequences) are shown. Mutation positions are numbered according to the reference Fermon strain (GenBank accession no. AY426531) and annotated by the corresponding nonstructural viral proteins (3A and 3D). For each of the positions showing a mutation in the new group, the frequency of each amino acid was visualized using WebLogo (https://weblogo.threeplusone.com). A, alanine; I, isoleucine; T, threonine; Y, tyrosine; V, valine.

**Figure 7:**
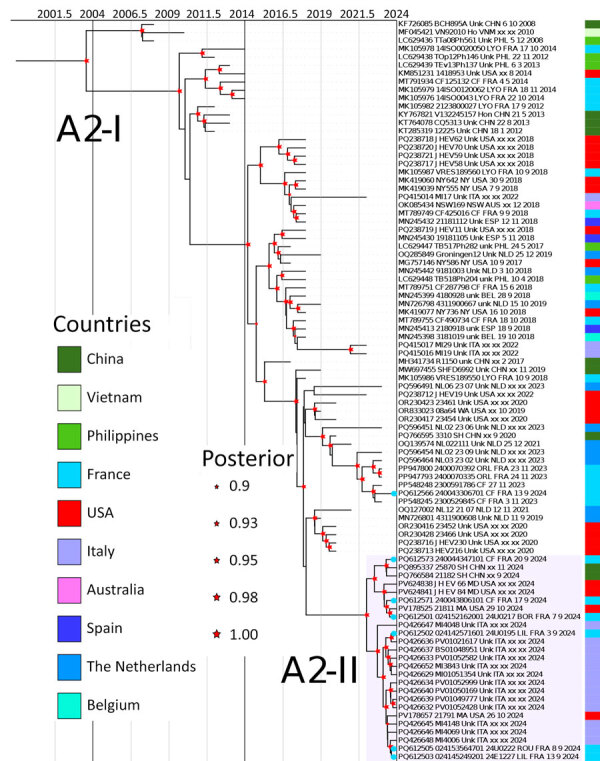
Bayesian time-scaled phylogeny of enterovirus D68, subgenotype A2, from report on national surveillance of enterovirus D68 upsurge, France, 2024. Phylogeny created using BEAST version 2.6.3 (https://beast.community). Subtree focusing on the most recent strains of subgenotype A2 was extracted from the complete time-calibrated phylogenetic tree. The estimated ages of the most recent common ancestors from the root of each clade to the year 2024 are indicated. Red stars at nodes indicate posterior probabilities >0.9, star sizes are proportional to posterior probability (larger symbols indicate values closer to 1). Colors at right indicate country of origin for each strain. Blue dots indicated at the taxon names indicate sequences generated in this study in 2024. GenBank accession numbers are provided.

**Figure 8:**
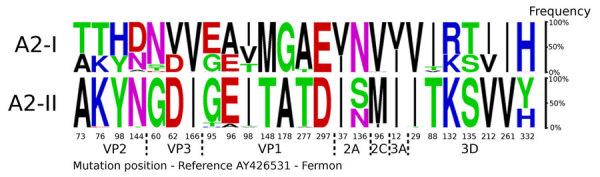
Amino acid change analysis of enterovirus D68, subgenotype A2, from report on national surveillance of enterovirus D68 upsurge, France, 2024. Amino acid substitutions leading to changes in the predominant residues between the newly identified A2-II group (n = 50 sequences) and the previously circulating A2-I (n = 75 sequences) clade are shown. Mutation positions are numbered according to the reference Fermon strain (GenBank accession no. AY426531) and annotated by the corresponding viral proteins (structural proteins VP2, VP3, and VP1 and nonstructural proteins 2A, 2C, 3A, and 3D). A, alanine; D, aspartate; E, glutamate; G, glycine; H, histidine; I, isoleucine; K, lysine; M, methionine; N, asparagine; R, arginine; S, serine; T, threonine; V, valine; VP, viral protein; Y, tyrosine.

**Figure 9:**
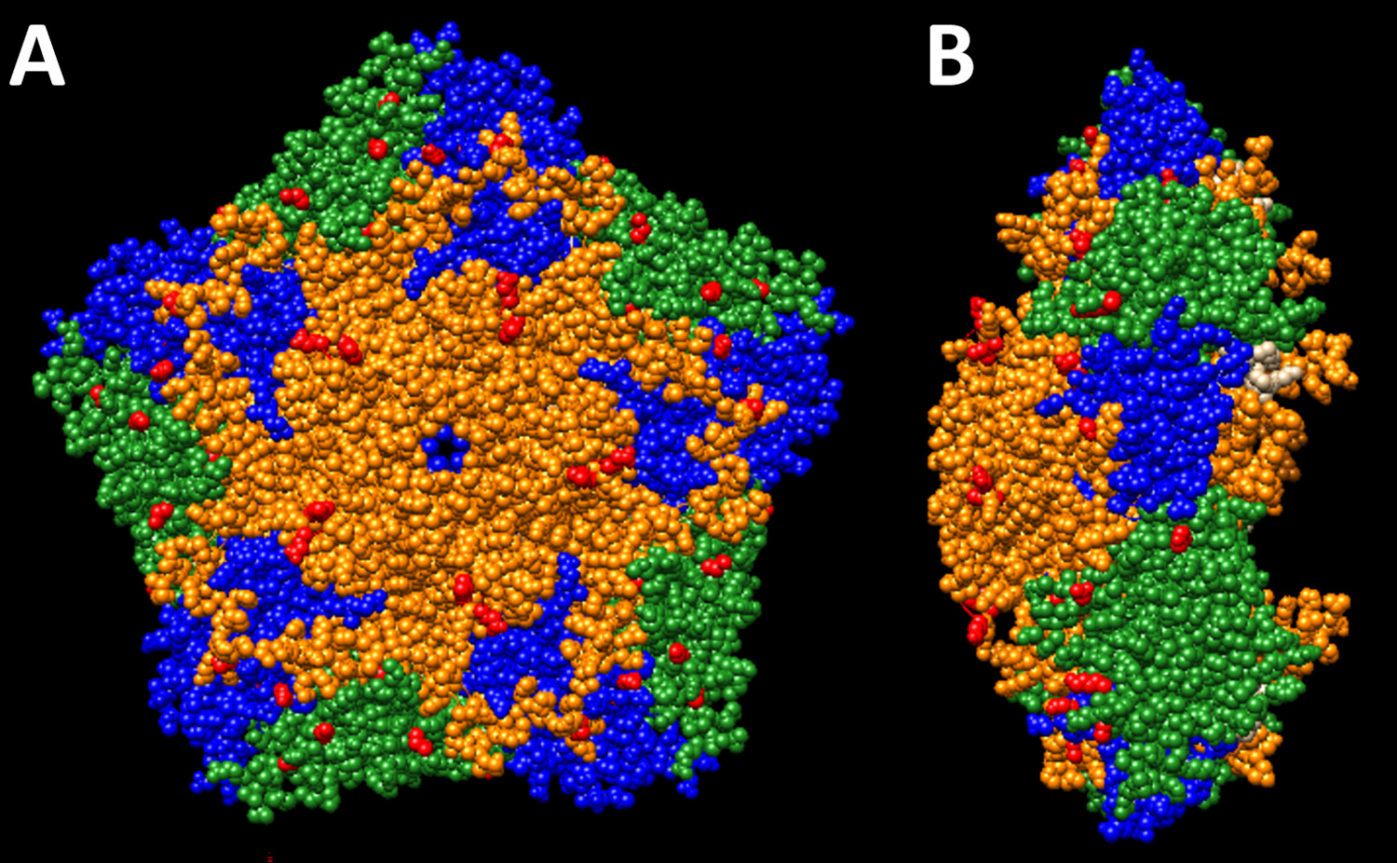
Structural models of the capsid pentamer of the newly identified A2 clade of enterovirus D68, from report on national surveillance of enterovirus upsurge, France, 2024. On the basis of a consensus sequence of the newly identified A2 clade, we reconstructed pentameric capsid subunit using AlphaFold (https://alphafoldserver.com) and validated it by structural comparison with the crystal structure of the reference enterovirus D68 strain (structure 4WM8; RCSB Protein Data Bank, https://www.rcsb.org). A) Outer surface view. B) Side view**.** Structural models of the capsid pentamer are shown with proteins colored as follows: orange, viral protein (VP) 1; green, VP2; blue, VP3; white, VP4. Red indicates amino acid substitutions.

Phylogenetic analysis of 449 near-complete coding sequences showed that most of the 2024 sequences clustered into 2 defined, well-supported groups, designated A2-II and B3-II in this study, distinct from other sequences among subgenotypes A2 and B3 (Figure 4). B3-II included most of the study sequences and recent 2024 outbreak strains from the United States and Italy and 2023 strains from the Netherlands, Senegal, and France (Figure 5). It likely emerged around early 2021 (time to most recent common ancestor 2021.2 [95% high posterior probability 2020.7–2021.6]) and is distinct of a broader B3 lineage, B3-I, mainly composed of strains collected in 2022–2023 worldwide. Three amino acid changes distinguished the B3-II group from B3-I group (Figure 6), all located outside the capsid region. The group A2-II formed a new A2 lineage comprising only sequences from 2024 strains collected mostly in France and Italy (*9*) (Figure 7). Time to most recent common ancestor was estimated at 2022.1 (95% high posterior probability 2021.2–2022.6); it shared a common ancestor with a lineage composed primarily of strains collected since 2019 in Europe and in the United States. That A2-II lineage exhibited 4.4% nucleotide and 1.4% amino acid divergence; 25 amino acid positions shifted in predominant residues of several proteins, compared with ancient A2 strains (Figure 8). In 15/25 positions, the dominant amino acids in 2024 strains (>90% prevalence) were previously minor variant in historical A2 sequences. Mutations in VP1 were located in key structural regions: the BC loop (residues 90–103), DE loop (110–169), GH loop (178–218), and the C-terminal domain (277–311) (*24*). Structural mapping onto a capsid pentamer model revealed that these capsid mutations are surface exposed (Figure 9, panels A, B).

## Discussion

We report the 2024 EV-D68 upsurge in France, which was characterized by an early onset, with the first consecutive weekly cases detected at the beginning of summer, and the highest number of cases detected by hospital-based surveillance since the reappearance of this virus in 2014. Those features can be partly explained by the enhanced laboratory surveillance and the intensive participation of the ESN, which sent 11 times more samples in 2024 than in 2021–2023. However, we observed higher EV-D68 positivity rates in 2024 than in 2021–2023 in both NRLs; each laboratory applied comparable strategies over the period. The adult population was particularly affected, as already observed for the EV-D68 surge reported in Italy in 2024 compared with 2023 (*9*). 

US multimodal surveillance of EV-D68 infections showed that the syndromic surveillance for asthmatic illness provided the best estimate of EV-D68 disease burden because of continued limited and selective sampling and testing for EV-D68 (*6*). However, that approach focused mainly on the pediatric population (*13*). In a Europe-based study, the combination of clinical enterovirus and respiratory surveillance was the most effective for detecting EV-D68 cases (*11*). In our study, year-round systematic screening for enterovirus/rhinovirus in respiratory samples using a diagnostic method capable of detecting EV-D68, followed by genotyping of positive samples, enabled the timely detection of EV-D68 upsurge. Of note, that surveillance approach, even when implemented across a limited number of laboratories, could therefore trigger an alert to the public health authorities. 

It is also important to raise clinician awareness about the need to reinforce EV-D68 screening not only in children but also in adults, particularly those with underlying or severe conditions, to enable a more accurate assessment of the effect of EV-D68 on this often underrecognized population (*4*,*25**,**26*). It has been widely reported that children are at greater risk of developing severe forms of EV-D68 infections (*3*,*5*); however, the resurgence observed in France in 2024 was associated with significant illness in adults. In the 2024 outbreak, 25.8% of adults required admission to intensive care departments, similar to the rate among children (24.8%). Regarding neurologic complications associated with EV-D68 infections, 7 (1.9%) children and 4 adults (1.4%) experienced severe neurologic signs; none of the children had signs of AFM but they did have seizures and signs of encephalitis. Among EV-D68 cases reported by 13 countries in 2021–2022 in Europe, 43 patients (6.8%) displayed neurologic disorders and half of those were diagnosed with seizures, encephalitis, meningitis, or AFM (*8*). The decrease in incidence of neurologic complications was also observed in the United States, where EV-D68 upsurges have not been associated with resurgences of myelitis since 2019, despite an effective surveillance of AFM cases (*6*,*7*). Since 2019, circulating EV-D68 viruses might be less neurotropic or less likely to cause neurologic disease than were viruses from 2014, 2016, and 2018 (*11*). All mutations, except for VP1:D283E, which was previously associated with neurovirulence traits (*27*), were absent in 2024 strains, including those of subgenotype A2 associated with the 4 adult cases of myelitis. Three of those adult cases were reported to be GBS. Because the AFM criteria are mainly based on pediatric cases (*7*,*28*) and that distinction between AFM and GBS might be challenging (*27*) particularly in adults, it is relevant to retain a broad and nonspecific definition such as acute flaccid paralysis, for the surveillance of EV-D68–associated myelitis.

B3-derived lineages were predominantly associated with recent EV-D68 outbreaks in Europe; an A2-derived lineage dominated the second part of the 2024 outbreak and was detected elsewhere in Europe, in North America, and in Asia. Since 2016, the main circulation of clade A2 in France had been observed only in 2018 (51% of EV-D68 infection), mainly in adults (*4*). Despite a new circulating lineage, the previously reported association of infection by the A2 subgenotype with adult patients (*4*,*29*) remained true in 2024 (*12*). Genomes of 2024 A2 viruses were genetically close to strains that simultaneously circulated in Italy, where the median age of cases with EV-D68 A2 lineage was 10-fold higher than that of cases with B3 subgenotype (*9*). Extensive genomic surveillance demonstrated periodic replacement of B3-derived lineages between epidemics and rapid renewal of epitopes in capsid proteins (particularly the BC and DE loops, and C-terminal end of the VP1 protein), potentially associated with antigenic evolution (*1*,*8*,*10*–*12*,*29*). Meanwhile, evolution of A2 viruses was also ongoing (*11*,*12*); numerous amino acid mutations accumulated that concentrate in VP1 surface loops and adjacent VP2 and VP3 surfaces, where neutralizing epitopes and receptor interaction cluster (*15*,*29*). In addition to functional implications, such as changes in receptor binding and increased transmissibility, those mutations could also have immunologic consequences that could explain the age–subgenotype association (*4*,*9*,*29*,*30*). The amino acid changes in the surface of recent A2 viruses could reduce cross-clade immunity (*31*) and foster reinfection of adults. Seroprevalence studies in adults that include strains from different subgenotypes are needed to determine the level of immunity to different and recent viruses. Adults could also be more susceptible to infection because of underlying conditions or specific mutations elsewhere in the viral genome promoting more severe infection or inflammation, leading them to seek medical attention.

The continued and sustained circulation of EV-D68 in recent years underlines the importance of comprehensive genomic surveillance of EV-D68 to track the spread of different subgenotypes and to understand their geographic distribution; Nextclade (https://github.com/enterovirus-phylo/nextclade_d68) is one useful tool for EV-D68 analysis (*29*,*32*). Adequate virologic and clinical surveillance, regardless of age and season, is essential to give a complete picture of the epidemiologic and clinical spectrum of EV-D68 and other emerging enterovirus type–associated infections.

Appendix Additional information from national surveillance of enterovirus D68 upsurge, France, 2024.
