# Supplementary material for: National Surveillance of Enterovirus D68 Upsurge, France, 2024
Source: Emerg Infect Dis. 2026 Jul;32(7):1082–93. doi: 10.3201/eid3207.260044 (PMC13322448; doi:10.3201/eid3207.260044)
Supplement: Appendix — Additional information from national surveillance of enterovirus D68 upsurge, France, 2024. [file 26-0044-Techapp-s1.pdf]

Article DOI: https://doi.org/10.3201/eid3207.260044

EID cannot ensure accessibility for supplementary materials supplied by authors. Readers who have difficulty accessing supplementary content should contact the authors for assistance.

National Surveillance of Enterovirus D68 Upsurge, France, 2024

Appendix

Reverse transcription and amplification protocols of the complete genome sequencing of EV-D68 in the National Reference Laboratory 1 (NRL1, Clermont-Ferrand, France).

In NRL1, complete genome sequencing was done after amplification of full-length genome. For reverse transcription, 5.5 µL of extracted RNA was incubated at 65°C for 5 minutes with 0.5 µL of the reverse primer polyT3NC_EVD68_R (5′-TTTTTTTTTTTTTTTTTTTTTTTTTGGYCCCAAGTGRCCAA AATTTACCTC-3′, 2 µM) and 0.5 µL of dNTPs (10 mM), then held at 4°C. Subsequently, 0.5 µL of SuperScript IV reverse transcription (200 U/µL; Thermo Fisher Scientific), 0.5 µL of RNaseOUT (40 U/µL; Thermo Fisher Scientific), 0.5 µL of DTT (0.1 M), and 2 µL of 5× reaction buffer were added to the reaction mix, yielding a final volume of 10 µL. Reverse transcription was performed at 55°C for 30 minutes, followed by enzyme inactivation at 80°C for 10 minutes. For near full-length genome amplification, 2 µL of cDNA was mixed with 6 µL of nuclease-free water, 10 µL of 2× SuperFi II PCR Master Mix (Thermo Fisher Scientific), and 1 µL each of the forward primer IFT7 EVD68_S (5′-GACAGCTTATCATCGTAATACGACTCACTATAGGGTTAAAACAGCCTTGGGGTTG-3′) and reverse primer R3D-EVD68 (5′-CGTCTAAGACTAGARTATGCAGGTAGTG-3′). The thermal cycling protocol was as follows: initial denaturation at 98°C for 1 minute; 41 cycles of denaturation at 98°C for 5 seconds, annealing at 60°C for 10 seconds, and extension at 72°C for 4 minutes and 30 seconds; followed by a final extension at 72°C for 5 minutes. To amplify the 3′ terminal region of the genome (~150 nt), cDNA synthesis was done as described above, using the reverse primer M13-dT18_R (5′-CAGGAAACAGCTATGACCGTTTTTTTTTTTTTTTTTT-3′). PCR amplification was then performed using SuperFi I DNA polymerase (Thermo Fisher Scientific), the forward primer

EVD68_7029_S (5′-ATTAGTAATGACACCAGC-3′), and the reverse primer M13-dT18_R, under the following cycling conditions: 98°C for 1 minute; 41 cycles of 98°C for 5 seconds, 55°C for 10 seconds, and 72°C for 20 seconds; followed by a final extension at 72°C for 5 minutes.

Sequencing libraries were prepared using the Illumina DNA Prep kit according to the manufacturer's instructions. Sequencing was carried out on the Illumina NextSeq 550 platform using a MidOutput flow cell (2×75 bp paired-end reads). Raw sequencing reads were demultiplexed using **bcl2fastq** (v2.20.0.422), and quality was assessed using **FastQC** (v0.11.9). Adaptor trimming and quality filtering were performed with **Trimmomatic**, and de novo assembly was conducted using **SPAdes** (v3.15.3).

Seven amplicons were sequenced with Oxford Nanopore technology as previously described (Mirand et al., 2025; doi: 10.1080/22221751.2025.2525266).

Appendix Table. EV-D68 subgenotype proportions among children and adults, France, 2024

EV-D68 infections with known subgenotype	Subgenotype B3, no. (%)	Subgenotype A2, no. (%)	Total
Children	227 (61.8)	140 (38.2)	367
Adults	37 (10.5)	317 (89.5)	354
Total	269 (36.7)	452 (63.3)	721
